# A wood density and aboveground biomass variability assessment using pre-felling inventory data in Costa Rica

**DOI:** 10.1186/s13021-014-0009-y

**Published:** 2014-09-17

**Authors:** Sienna Svob, J Pablo Arroyo-Mora, Margaret Kalacska

**Affiliations:** Department of Geography, McGill University, Burnside Hall Building, Montreal, H3A 0B9 QC Canada

**Keywords:** Forest management, Aboveground biomass, Wood density, Tropical forest, Costa Rica

## Abstract

**Background:**

The high spatio-temporal variability of aboveground biomass (AGB) in tropical forests is a large source of uncertainty in forest carbon stock estimation. Due to their spatial distribution and sampling intensity, pre-felling inventories are a potential source of ground level data that could help reduce this uncertainty at larger spatial scales. Further, exploring the factors known to influence tropical forest biomass, such as wood density and large tree density, will improve our knowledge of biomass distribution across tropical regions. Here, we evaluate (1) the variability of wood density and (2) the variability of AGB across five ecosystems of Costa Rica.

**Results:**

Using forest management (pre-felling) inventories we found that, of the regions studied, Huetar Norte had the highest mean wood density of trees with a diameter at breast height (DBH) greater than or equal to 30 cm, 0.623 ± 0.182 g cm^-3^ (mean ± standard deviation). Although the greatest wood density was observed in Huetar Norte, the highest mean estimated AGB (EAGB) of trees with a DBH greater than or equal to 30 cm was observed in Osa peninsula (173.47 ± 60.23 Mg ha^-1^). The density of large trees explained approximately 50% of EAGB variability across the five ecosystems studied. Comparing our study's EAGB to published estimates reveals that, in the regions of Costa Rica where AGB has been previously sampled, our forest management data produced similar values.

**Conclusions:**

This study presents the most spatially rich analysis of ground level AGB data in Costa Rica to date. Using forest management data, we found that EAGB within and among five Costa Rican ecosystems is highly variable. Combining commercial logging inventories with ecological plots will provide a more representative ground level dataset for the calibration of the models and remotely sensed data used to EAGB at regional and national scales. Additionally, because the non-protected areas of the tropics offer the greatest opportunity to reduce rates of deforestation and forest degradation, logging inventories offer a promising source of data to support mechanisms such as the United Nations REDD + (Reducing Emissions from Tropical Deforestation and Degradation) program.

**Electronic supplementary material:**

The online version of this article (doi:10.1186/s13021-014-0009-y) contains supplementary material, which is available to authorized users.

## Introduction

Tropical forests play a vital role in regulating the Earth's climate through the processes of evapotranspiration and CO_2_ uptake. While these areas represent only 7% of global land cover [1], they store roughly 55% of the world's forest carbon stock [2]. Tropical forests are among the most carbon dense ecosystems (242 Mg C ha^-1^) in the world [2]. Approximately 56% (193-223 Pg C) of their carbon is stored in the form of biomass alone [2]-[4]. During the 1990s and early 2000s, a substantial portion of this carbon stock suffered due to deforestation, which reached an estimated rate of 12.9•10^6^ ha yr^-1^[5]. The deforestation and degradation of tropical forests is also the second largest source of anthropogenic CO_2_ emissions [6], releasing carbon at an estimated net rate of 1.0 Pg yr^-1^ between 2000 and 2010 [4].

The United Nations REDD + (Reducing Emissions from Deforestation and Forest Degradation) program is an innovative global mechanism that aims to provide monetary benefits to developing tropical countries that can show an increase in forest carbon stocks from an established national baseline [7]. In the past decade, the number of studies seeking to improve the methods and data used to accurately estimate the spatio-temporal variation of tropical forest carbon stocks, supporting REDD+, have substantially increased [8]. Today, much of this research relies upon the relationship between aboveground biomass (AGB) and forest carbon. Despite recent efforts to estimate AGB in the tropics (and in turn carbon) (refer to [3],[4],[9]), a large degree of uncertainty in the spatial distribution and accuracy of these estimates remains [10],[11]. One of the key factors in reducing uncertainty in AGB estimates is using a spatial scale fine enough to capture variability across the landscape.

Remote sensing and ground data (i.e. forest inventories) are two techniques that have been proposed for the production of reliable carbon estimates (e.g. [12]). Remote sensing is an advantageous approach as it can provide wall-to- wall coverage of an entire country. Remotely sensed data, however, must be calibrated/validated with ground truth measurements [13],[14]. Additionally, remote sensing instruments may not be sensitive enough to detect the variability of biomass within and across the high-density forest stands [14],[15] typical of tropical moist and tropical wet ecosystems. Ground data collected for scientific research (ecological data), is the most common data source employed to estimate AGB due to its high level of detail and systematic nature. Nevertheless, ecological data has its own weaknesses when estimating AGB such as: (1) the standard plot size of 0.1 ha [16] is too small to capture AGB variability [17]; (2) plots are sometimes biased towards high density (ideal) forest locations [16]; and (3) plots cover only a small fraction of a country's total forested area [18]. Commercial logging inventories may provide a solution to these problems due to their spatial distribution and sampling intensity [18],[19]. Logging inventories are common to tropical countries and represent a large source of data on forest structure and composition [20]. With their success in measuring diversity on large spatial scales [21], determining ecological factors that influence forest structure [19], and estimating emission factors under REDD + [18], logging inventories may provide a suitable source of forest data, complementing ecological datasets and helping to estimate baseline carbon stocks.

In Costa Rica, selective logging inventories or pre-felling inventories (we use this terminology in the paper indistinctively) are developed under a Natural Forest Management Plan (NFMP) framework [22]. NFMP data is available for most of the country's ecosystems below an elevation of 300 meters, accurately representing the heterogeneity of the Costa Rica's lowland landscape. The country is divided into 11 conservation areas (CAs) [23], each encompassing distinct forest ecosystems. Despite the country's small size (approximately 51 000 km2), it contains a rich diversity of tropical ecosystems ranging from dry to wet forests.

A NFMP is required before the forest stand of a privately owned property can be selectively logged. In order to be legally approved to log, the owner must hire a certified forester to conduct an inventory and census of the forest stand [22]. In an inventory, every tree with a diameter at breast height (DBH) equal to or greater than 30 cm is measured and identified in plots of 0.3 ha. For the same forest stand, a census is carried out to measure and identify every tree with a DBH equal to or greater than 60 cm. For this study we use a standardized relational geodatabase encompassing Costa Rican pre-felling inventory data [24].

The main objective of this study is to assess to variability of wood density and estimated AGB across five ecosystems in Costa Rica. Wood density is an important predictive variable when estimating AGB [16],[25]-[27]. As wood density is known to vary among different forest communities [28]-[31], this variable is also critical to studying the differences in AGB across a landscape. Despite these findings, wood density has yet to be studied or implemented when estimating AGB across Costa Rica. Further, although our analysis is based on medium to large trees (30 and 60 cm DBH), studies have shown that large trees constitute a disproportionate fraction of AGB and drive the variations in biomass across the tropics [32]. Therefore, despite a lack of tree data below the 30 cm DBH range, patterns of AGB variability may be discernible from our NFMP dataset. A standard method to estimate a tree's biomass employs an allometric equation to relate measurements on DBH to units of biomass. The choice of allometric model is critical and should be based upon both the aim of the study [28] and the characteristics of the dataset [33]. Allometric models should be representative of the DBH range and ecosystem being studied [33]. Additionally, to allow for regional scale comparisons, AGB estimates must be based on a consistent regression approach to avoid the confounding of results by variations inherent in different models [28].

In recent years, the pantropical allometric models developed by Chave et al. [25] have been widely applied across the globe to estimate AGB. In Costa Rica, however, numerous studies of biomass have employed Brown's [34] equation for wet forests (e.g. [17],[35],[36]) as it was calibrated with data collected at Costa Rica's La Selva Biological Station. For this study, we believe Brown's [34] equation has many disadvantages when compared to those developed by Chave et al. [25]. These shortcomings include: (1) the representation of a smaller DBH range; (2) the development of the equation from a smaller sample size; (3) the limited application of the equation outside of Costa Rica (making the comparison of AGB estimates with other countries/studies more complex); and (4) the absence of wood density as a parameter which is an aspect of forest structure that varies significantly at regional scales [28],[30].

With the use of allometric models [25], the Global Wood Density database [30],[37], the pre-felling inventory database [24], and national measurements of wood density found in the scientific literature (e.g. [38]), this study will first, evaluate the variability in wood density and second, assess the variability in estimated AGB across five ecosystems in Costa Rica and different sampling protocols. Specifically, our study uses a NFMP database for five conservation areas to address the following questions:

1. What are the patterns of wood density variability at the CA-level and between data produced by the census and inventory (i.e. sampling protocols)?;
2. What is the variability of estimated AGB within and among CAs?;
3. Do estimated AGB values differ between the two sampling protocols?
4. What is the uncertainty associated with AGB estimated using natural forest management data?

As ground level data from pre- felling inventories covers a greater area than ecological plots within the five ecosystems being studied, our study will better capture the spatial heterogeneity of wood density and estimated AGB across the landscape. Through this analysis, we can enhance our understanding of the spatial distribution of estimated AGB and, in combination with both ecological and remotely sensed data, more reliably map and estimate national forest carbon stocks.

## Methods

### Study area and data

**Figure 1:**
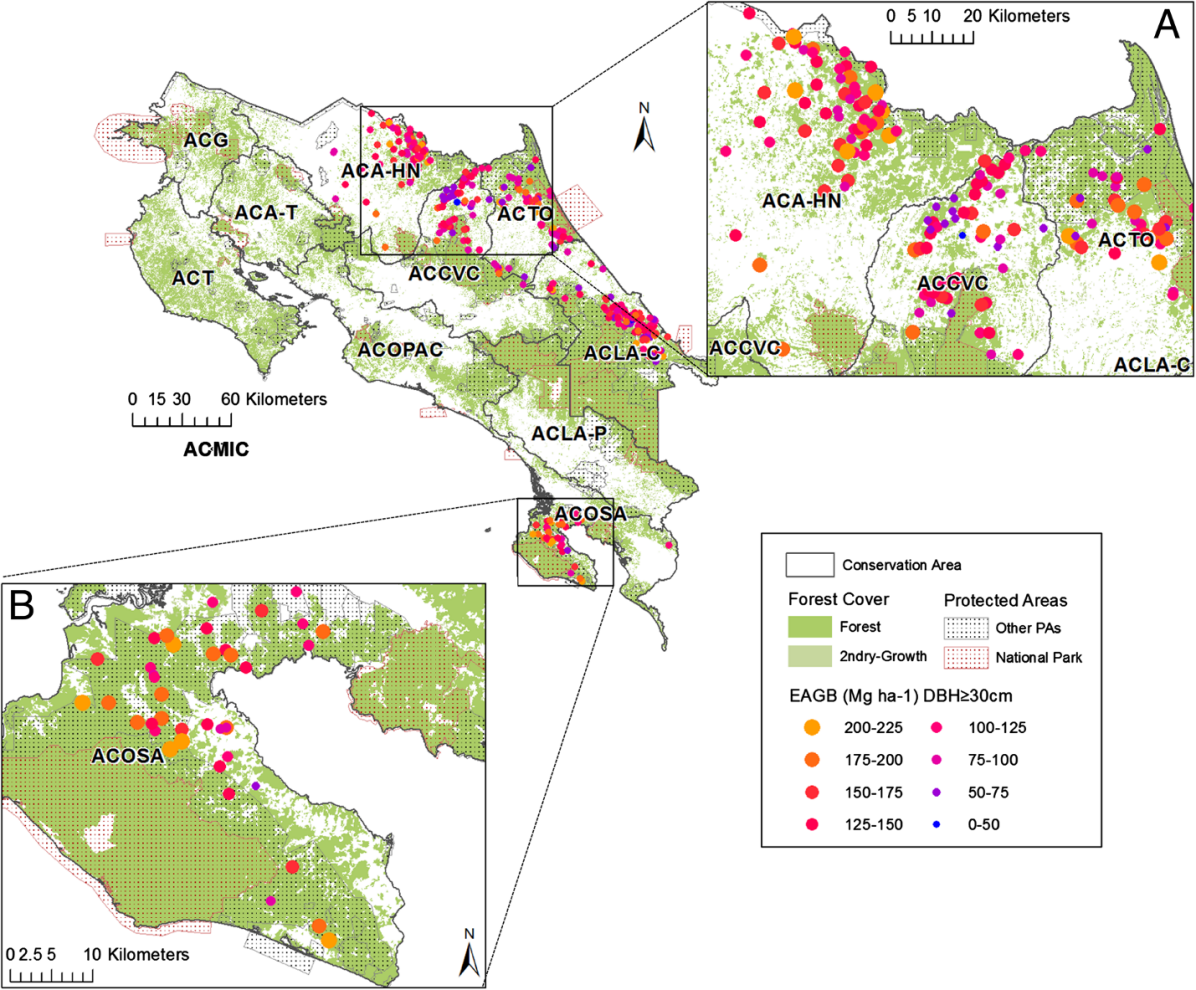
**Map of the EAGB (Mg ha**^**-1**^**) of trees with a DBH ≥ 30 cm per NFMP. (A)** A view of the distribution NFMPs collected in ACAHN, ACCVC, and ACTO. **(B)** A view of NFMPs collected in ACOSA. Forest cover source: FONAFIFO 2005 Costa Rica forest cover assessment. Protected areas sources: Atlas of Costa Rica 2008 [41].

This study used a database of NFMPs from five conservation areas: ACLA-C (Caribbean La Amistad Conservation Area), ACAHN (Arenal Huetar Norte Conservation Area), ACTO (Tortuguero Conservation Area), ACCVC (Central Volcanic Conservation Area), and ACOSA (Osa Conservation Area) (Figure 1). Greater detail about the NFMP database and the taxonomic, wood density, spatial, and tree measurement data used in this study is provided in Svob et al. [24]. The differences found between the historical patterns of natural forest management of the five conservation areas, such as the number of NFMPs per conservation area, forest fragmentation statistics specific to each conservation area, and descriptive statistics based on the NFMPs collected in each conservation area, are given in Arroyo-Mora et al. [39]. The five conservation areas considered in this study cover the country's Atlantic lowland forests, northern lowlands, and central and south Pacific forests, encompassing the regions where selective logging has been most heavily practiced. All five conservation areas include natural forest management plans that fall within the tropical wet (4000-8000 mm precipitation yr^-1^) and/or rain (>8000 mm precipitation yr^-1^) forest lifezones (defined by Holdridge [40]). Only ACLA-C and ACAHN include natural forest management plans that represent the tropical moist (2000-4000 mm precipitation yr^-1^) forest lifezone. The management plans sampled largely represent a lowland ecosystem (0-500 m a.s.l.) although a small subset of the data falls within the transition zone from lowland to premontane (500-1500 m a.s.l.) forest. The forest type of the natural forest management plans was classified using the Life Zone System Map from the Atlas Costa Rica 2008 [41]. All management plans were carried out in primary forest (old growth).

### The variability of wood specific gravity among CAs and sampling protocols

The variability of wood density across Costa Rica can be illustrated by differences found in wood density between conservation areas, NFMPs, and the different sampling protocols (census and inventory). The greater the variability in wood density, the more important it becomes to include this parameter when producing AGB estimates comparable at a landscape-scale. To carry out the analysis, we excluded data from NFMPs with less than 80% of their trees identified to the species or genus level. The wood density value for each tree in a NFMP was selected in decreasing order of preference from (1) a species-level average, (2) a genus-level average, and (3) a NFMP-level average. Mean NFMP wood density was calculated separately for each census and inventory. Wood density values were extracted from the NFMP database and are described in greater detail in Svob et al. [24]. These NFMP averages were determined by summing the wood density of all stems with a species or genus level value. Differences between the wood density of the conservation areas and the two different sampling protocols were tested with a one-way ANOVA and a multiple comparisons procedure using the grouping variables conservation area and sampling protocol. All conservation area level statistical analyses only included tree data that met the aforementioned NFMP taxonomic identification conditions. All of the analyses were carried out in MATLAB version R2013b (The MathWorks Inc., Natick, MA, USA) unless otherwise specified.

### Estimating tree-level AGB

In this study, we denote estimated AGB as estimated aboveground biomass (EAGB), following Clark and Kellner [10]. To determine EAGB per tree, Chave et al.'s [25] allometric models for wet and moist forests were applied. The EAGB of wet and rain forest lifezones was estimated by applying the wet forest equation:

1 $$\text{EAGB}=\rho \times \exp\left(-1.239+1.980\times \ln\left(\text{DBH}\right)+0.207\times \ln{\left(\text{DBH}\right)}^{2}-0.0281\times \ln{\left(\text{DBH}\right)}^{3}\right.$$

Correspondingly, the EAGB of forests within the tropical moist lifezones was estimated using the moist forest equation:

2 $$\text{EAGB}=\rho \times \exp\left(-1.499+2.148\times \ln\left(\text{DBH}\right)+0.207\times \ln{\left(\text{DBH}\right)}^{2}-0.0281\times \ln{\left(\text{DBH}\right)}^{3}\right.$$

where ρ is wood density in g cm^-3^, DBH is in cm, and EAGB is given in kg tree^-1^. Wood density values were selected in decreasing order of preference from (1) a species-level average, (2) a genus-level average, (3) a NFMP-level average, and (4) a conservation area level average. A large portion of the variation in wood density is captured at the genus-level, making mean genus wood density the second best option when estimating EAGB [28],[30]. We use the mean NFMP wood density for a tree that was present in a NFMP with at least 80% of its trees identified to the genus or species level and was missing a corresponding species or genus level wood density. For a tree that was reported in a NFMP with less than 80% of its trees identified to the species or genus level and lacked wood density information, we used a conservation area level mean wood density.

### Estimating census and inventory AGB

To calculate EAGB per unit area of a census, the EAGB values per tree with a DBH greater than or equal to 60 cm were summed and then divided by the productive area of the NFMP's forest stand. The productive area is the total area of the forest stand sampled by a census. To calculate EAGB per unit area of an inventory, the EAGB values per tree with a DBH greater than or equal to 30 cm were summed across each plot and then divided by 0.3 ha (area of the plot). Finally, EAGB was averaged across all plots within a given NFMP.

An outlier analysis of all of the resulting EAGB values was performed, pinpointing cases where EAGB did not fall between the 1.5 lower and 1.5 upper interquartile range. All outliers were cross-checked with the original hardcopy versions of the NFMPs. If the values were the result of uncorrectable errors present in the original NFMPs, they were excluded from any further analyses. To assess the amount of spatial autocorrelation among the NFMPs sampled, we evaluated the distribution of Moran's I with a spatial correlogram as applied in SAM version 4.0. Spatial correlograms indicate the correlation between pairs of spatial observations as the distance between them is increased [42]. As the values of Moran's I were relatively small, ranging between 0.154 and -0.209, we did not include any additional methodological approaches to account for spatial autocorrelation in later analyses.

### Comparing the EAGB of the sampling protocols and CAs

Differences in EAGB between conservation areas and between sampling protocols (tree census vs tree inventory) were tested with a one-way ANOVA and a multiple comparisons procedure using conservation area and sampling protocol as the grouping variables. We also evaluated differences in the density of large trees (DBH ≥ 70 cm) between CAs and sampling protocols, as the recent study of Slik et al. [32] found that large trees explained up to 70% of the variation in EAGB across the tropics.

In order to compare the data captured by inventories and censuses more directly, EAGB was recalculated for each inventory including only the trees that would be sampled during a census (DBH ≥ 60 cm). To assess whether there was a significant difference in this data, a paired *t*-test was applied. As multiple NFMPs only included census data (DBH ≥ 60 cm), we attempted to develop a model to estimate the EAGB of stems with a DBH ≥ 30 cm and a DBH < 60 cm. We attempted to develop a model by relating, for each pre-felling inventory, the EAGB of trees with a DBH ≥ 60 cm to the EAGB of trees with a DBH ≥ 30 cm and a DBH < 60 cm. We compared the ability of a number of regression models (linear, exponential, logistic, and polynomial) to capture a relationship between EAGB (DBH ≥ 60 cm) and EAGB (60 cm < DBH ≥ 30 cm).

### Evaluating the uncertainty of AGB estimates

Uncertainty can be introduced to a tree's EAGB through DBH measurement errors (σMDBH), wood density measurement errors (σMρ), and errors inherent in the allometric model itself (σA) [33]. In this study, we evaluated the uncertainty of tree-level AGB estimates introduced by error in wood density measurements (σMρ) at the four following levels: species (σMρ:sp), genus (σMρ:gen), NFMP (σMρ:NFMP), and conservation area (σMρ:CA). We hypothesized that the measurement error will increase as the source of wood density increases in taxonomic scale. To evaluate errors introduced by the allometric models themselves, we reiterated the calculation of a tree's EAGB while varying ε (Equations 3 and 4) based on the residual standard error (RSE). Monte Carlo simulations were run in MATLAB version R2013b (The MathWorks Inc., Natick, MA, USA) to simulate the parameters (wood density and residual standard error of the allometric model (ε)) and determine both wood density measurement error and allometric model error.

As wood density values vary at the tree level within NFMPs, conservation areas, species, and genera [30], using mean wood density values to estimate AGB will introduce measurement error. Further, in forest management inventories, trees are identified in the field by common names and later related to scientific names. This methodology can result in the misidentification of species [43] and therefore, additional wood density measurement errors. We evaluate wood density errors under the assumption that the errors have a centered normal distribution. The distribution of errors for each tree uses the calculated mean and standard deviation of the appropriate species, genus, NFMP, or conservation area. We randomly selected 10 000 trees (5 000 from the census and 5 000 from the inventory) to evaluate the uncertainty at each level. For each tree, we calculated EAGB 1 000 times while varying the wood density parameter by a random normal distribution.

As allometric models are typically created using a regression on log-transformed variables, there is inherent error built in them. This uncertainty is the result of trees departing from the exact allometry described by the models [33]. Errors in tree AGB estimates due to the allometric model were assessed by varying ε (Equation 3 and 4) following the methodology of Maniatis et al. [18]. We assumed that ε followed a centered normal distribution with a mean of 0 and a standard deviation of 0.356 (residual standard error reported for the models in Chave et al. [25]). ε was incorporated into the EAGB equations using the same structure as Maniatis et al. [18]. For wet forests the model became:

3 $$\text{EAGB}=\rho \times \exp\left(-1.302+1.980\times \ln\left(\text{DBH}\right)+0.207\times \ln{\left(\text{DBH}\right)}^{2}-0.0281\times \ln{\left(\text{DBH}\right)}^{3}\right)\times \exp\left(\varepsilon \right)$$

while for moist forests is became:

4 $$\text{EAGB}=\rho \times \exp\left(-1.562+2.148\times \ln\left(\text{DBH}\right)+0.207\times \ln{\left(\text{DBH}\right)}^{2}-0.0281\times \ln{\left(\text{DBH}\right)}^{3}\right)\times \exp\left(\varepsilon \right)$$

Following the previously described methodology, 10 000 were randomly selected from the census and inventory and, for each tree, EAGB was calculated 1000 times while varying the ε parameter by a random normal distribution.

To evaluate the uncertainty of EAGB at the census and inventory levels, EAGB was simulated 1 000 times for every tree of 100 randomly selected censuses and 100 randomly selected inventories. For each simulation, we varied both wood density and ε simultaneously following the above sampling methodology. Simulated EAGB values were compared, revealing the uncertainty and precision of the reported EAGB values.

## Results

### The variability of wood density

**Figure 2:**
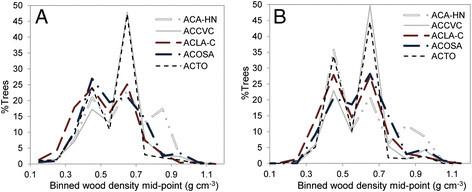
**The distribution of wood density within five CAs.** The percentage of trees that occupy consecutive 0.1 g cm^-3^ wood density bins in NFMP where **(A)** inventories (DBH ≥ 30 cm) and **(B)** censuses (DBH ≥ 60 cm) in the five CAs sampled.

Among all conservation areas, ACLA-C had a significantly lower wood density (inventory: 0.528 ± 0.161 g cm^-3^, p < 0.01; census: 0.530 ± 0.1520 g cm^-3^, p < 0.01) (mean ± standard deviation unless otherwise specified) (Table 1). No difference in wood density was detected in the census data among ACOSA, ACCVC, and ACAHN. Based on the inventory data set (either DBH ≥ 30 cm or only including DBH ≥ 60 cm), however, ACAHN had a significantly higher wood density than the other four conservation areas (0.623 ± 0.182 g cm^-3^ and 0.636 ± 0.197 g cm^-3^ respectively). The greater mean wood density found in ACAHN is due to a larger fraction of trees within the 0.8 to 0.9 g cm^-3^ range (Figures 2A and 2B). This is a result of the high density of the *Dialium guianensis* in ACAHN [24]. In ACCVC and ACTO, a prominent peak in the percent of stems within the 0.6 to 0.7 g cm^-3^ range can be attributed to the high relative frequency of *Pentaclethra macroloba*. The range of wood density values sampled was similar in all five conservation areas. Additionally, in all conservation areas, the mean wood density sampled by the inventory and that sampled by the census did not significantly differ.

**Table 1:** **Wood density (g cm** ^**-3**^ **) per conservation area and sampling protocol**

	Wood Density (g cm^-3^)		
	(Mean ± Std)		
CA	Inventory	Inventory	Census
	(DBH ≥ 30 cm)	(DBH ≥ 60 cm)	(DBH ≥ 60 cm)
ACAHN	0.623 ± 0.182	0.636 ± 0.197	0.602 ± 0.189
ACCVC	0.602 ± 0.144	0.579 ± 0.170	0.600 ± 0.143
ACLA-C	0.528 ± 0.161	0.579 ± 0.171	0.530 ± 0.152
ACOSA	0.574 ± 0.165	0.579 ± 0.169	0.604 ± 0.166
ACTO	0.565 ± 0.143	0.578 ± 0.169	0.564 ± 0.140

### The variability of EAGB

**Figure 3:**
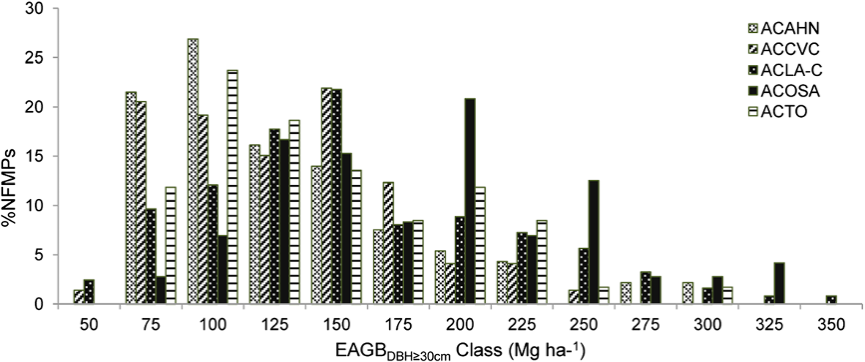
**The distribution of inventory EAGB (DBH ≥ 30 cm) per CA.** The distribution is shown by the percentage of NFMPs within 25 Mg ha^-1^ EAGB bins.

Based on inventory data, the estimated AGB (EAGB) (DBH ≥ 30 cm) found in ACAHN and ACCVC was significantly lower than that in ACLA-C and ACOSA (p < 0.05) (Figure 3 and Table 2). While ACAHN, ACCVC, ACTO, and ACLA-C all shared similar inventory EAGB values with at least one other conservation area, only in ACOSA did EAGB differ significantly from all other CAs. In fact, ACOSA presented the highest mean inventory EAGB (173.47 ± 60.23 Mg ha^-1^, p < 0.05).

**Table 2:** **EAGB (Mg ha** ^**-1**^ **) and large tree density (tree ha** ^**-1**^ **) per conservation area and sampling protocol**

	Inventory (Mean ± Std)			Census (Mean ± Std)	
CA	EAGB_DBH≥30 cm_	EAGB_DBH≥60 cm_	Lrg tree density	EAGB_DBH≥60 cm_	Lrg tree density
	(Mg ha^-1^)	(Mg ha^-1^)	(tree ha^-1^)	(Mg ha^-1^)	(tree ha^-1^)
ACAHN	118.07 ± 54.09	65.25 ± 33.81	6.29 ± 3.55	39.77 ± 23.48	4.25 ± 2.72
ACCVC	116.17 ± 44.48	63.35 ± 29.99	6.71 ± 4.02	45.22 ± 26.05	5.27 ± 3.39
ACLA-C	143.38 ± 62.18	79.83 ± 48.29	9.21 ± 5.98	53.60 ± 27.89	6.15 ± 2.99
ACOSA	173.39 ± 60.64	123.27 ± 53.56	16.30 ± 7.97	58.35 ± 23.24	7.65 ± 2.79
ACTO	130.30 ± 51.05	80.12 ± 39.21	10.70 ± 6.26	52.36 ± 31.46	6.98 ± 4.27

**Figure 4:**
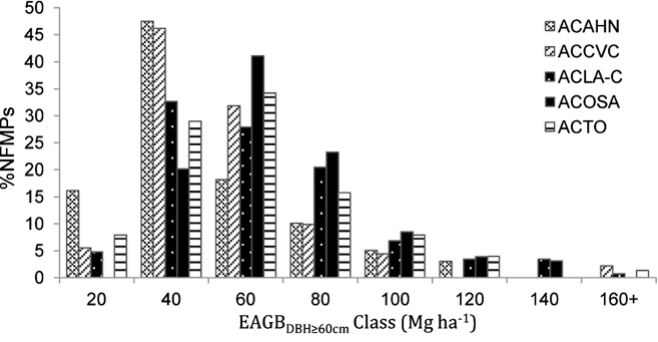
**The distribution of census EAGB (DBH ≥ 60 cm) per CA.** The distribution is shown by the percentage of NFMPs within 20 Mg ha - 1 EAGB bins.

**Figure 5:**
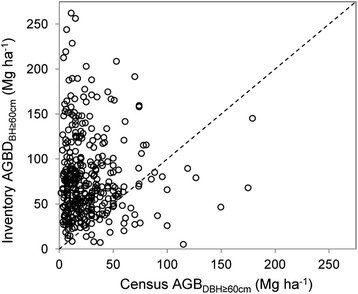
**Scatter plot of census EAGB (DBH ≥ 60 cm) versus inventory EAGB (DBH ≥ 60 cm).** The dashed diagonal line depicts a 1:1 relationship between census and inventory EAGB values.

Based on census data, ACAHN had the lowest EAGB, significantly differing from ACLA-C, ACTO, and ACOSA (p < 0.05) (Figure 4 and Table 2). Simply ranking conservation areas in decreasing order of mean EAGB (Table 2), reveals that the overall trends are similar between sampling protocols. For example, ACAHN had one of the lowest mean EAGB values in both the census and inventory data (39.77 ± 23.48 Mg ha^-1^ and 136.63 ± 60.08 Mg ha^-1^). A paired *t*-test comparing the EAGB of trees with a DBH ≥ 60 cm from the census and inventory detected a significant difference between the two sampling protocols (n = 366, p < 0.01). Across all five conservation areas, inventories generally produced higher EAGB values than censuses of the same forest stand (Figure 5).

**Figure 6:**
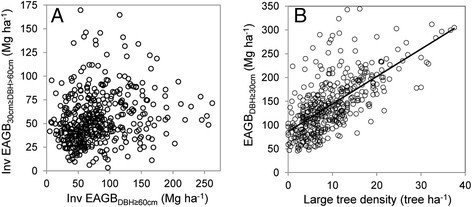
**The relationship between the components of forest structure for trees of different sizes. (A)** Scatter plot of inventory EAGB (60 cm > DBH ≥ 30 cm) versus inventory EAGB (DBH ≥ 60 cm). The distribution of points within the scatter plot reveals the variation found between these two EAGB DBH classes; **(B)** The relationship between the density of large trees (DBH ≥ 70 cm) and EAGB (DBH ≥ 30 cm). The line fit through the data explains 53.3% of the variation in EAGB.

Our attempt to relate the EAGB of trees with a DBH ≥ 60 cm to the EAGB of trees with a DBH ≥ 30 cm but <60 cm did not indicate a strong relationship between the two variables (e.g. linear regression results adj R^2^: 0.043, F = 19.9, p < 0.01, n = 422). Although we were unable to find a relationship between the two variables, this analysis demonstrates the amount of variance in the structure of Costa Rican forests (Figure 6A).

### The density of large trees (DBH ≥ 70 cm)

We found a significant correlation between the densities of large trees (DBH ≥ 70 cm) and EAGB (DBH ≥ 30 cm) (correlation coefficient: 0.728, n: 422, adj R^2^: 0.533, F = 470.6, p < 0.01) (Figure 6B). Our results show that 53.3% of the variation in EAGB across five Costa Rican conservation areas was explained by the density of large trees. Additionally, trends in the density of large trees per conservation area match trends in EAGB per conservation area. For example, in ACOSA, the EAGB and density of large trees (16.48 ± 8.08 tree ha^-1^) were both significantly higher than in the four other CAs (Table 1). Furthermore, the two conservation areas with the lowest mean EAGB (ACAHN and ACCVC) also had the lowest mean density of large trees (6.20 ± 3.54 and 6.73 ± 4.00 tree ha^-1^ respectively). Across all conservation areas, a pairwise *t*-test indicates a significantly higher number of large trees per hectare was recorded by the inventory than the census of the same forest stand (n = 366, p < 0.01).

### Uncertainty analysis

At the tree-level, when moving from species wood density (σMρ:sp = 0.110 〈EAGB**〉**) to genus wood density (σMρ:gen = 0.151 〈EAGB**〉**), we found a 4% increase in the uncertainty of EAGB due to wood density measurement error (σMρ). We found that an even greater amount of EAGB uncertainty resulted from using NFMP (σMρ:NFMP =0.271 〈EAGB**〉**) or conservation area (σMρ:CA =0.281 〈EAGB**〉**) level wood density values. The uncertainty due to allometric model error (σA) for each tree was 0.371 〈EAGB**〉**. Hence, based on our Monte Carlo uncertainty analysis, the uncertainty of a tree's EAGB can range from 48% to 65% of its EAGB depending on the level of wood density used.

**Figure 7:**
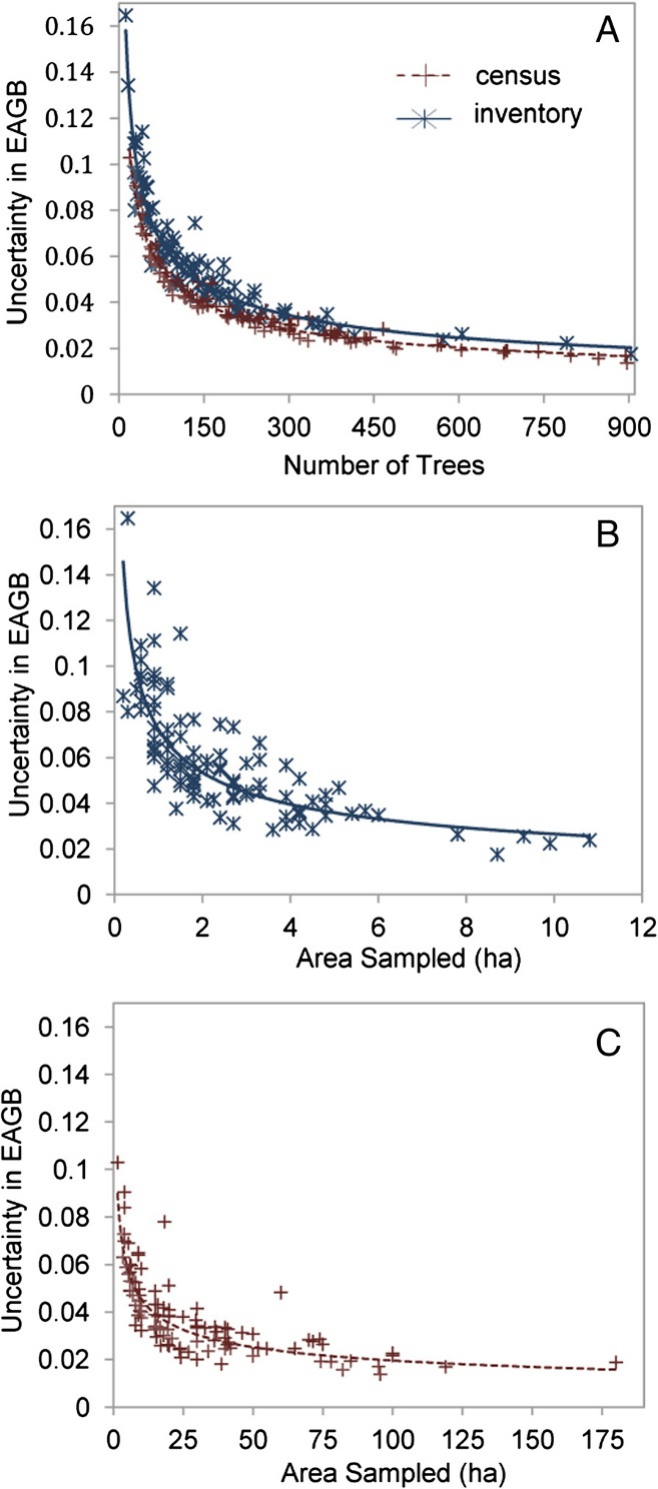
**The relationship between the uncertainty in EAGB per NFMP and sampling effort. (A)** The total number of trees sampled per NFMP; **(B)** the total area sampled per NFMP inventory; and **(C)** the total area sample per NFMP census. Uncertainty is given in unit-less value that varies from 0 to 1. The solid (inventory) and dashed (census) lines depict the best fit function (power).

At the stand level, random measurement and allometric model errors counteract one another, decreasing their impact on EAGB uncertainty and increasing the overall precision of EAGB (Figure 7). The uncertainty of EAGB from a NFMP's inventory ranged from 0.021 〈EAGB**〉** to 0.171 〈EAGB**〉**. At the census level, the uncertainty of EAGB for each NFMP ranged from 0.011 〈EAGB**〉** to 0.101 〈EAGB**〉**.

We observed that the uncertainty of a given NFMP's EAGB was principally dependent on the number of trees sampled and the total area sampled (Figure 7). In Figure 7, we also observed that the uncertainty of EAGB decreases as the number of trees (Figure 7A) and the total area sampled increases (Figure 7B and 7C) following a power function.

## Discussion

### The variability of wood density

Our study demonstrates for the first time the variability of wood density across five Costa Rican conservation areas based on pre-felling inventory data. We found the most northern forests included in our study, located in ACAHN, typically contain trees of higher wood density than those located in the other conservation areas sampled. In contrast, our results show that the southeastern lowland forests of ACLA-C house trees that tend to have lower wood density values. The regional differences between wood density values detected by our study indicate the importance of including this variable for the production of AGB estimates that are comparable at regional scales across Costa Rica. This variation also suggests that using more general country or pantropical scale wood density values when estimating AGB may lead to inaccurate results, underestimating the variability of EAGB across tropical regions [28],[29]. The regional wood density values found express the similarities and differences in species composition between the five conservation areas studied. Beyond species composition, it is also known that wood density is closely linked to a forest's functional composition as light-demanding fast-growing species commonly have lower wood densities than shade-tolerant ones [44],[45]. Building upon this idea, we believe that both natural and human disturbance regimes may play a key role in shaping the variation of EAGB and wood density among the forest stands and conservation areas studied.

### The variability of EAGB

The variation of EAGB among NFMPs within any given conservation area reveals the heterogeneity of EAGB across the five conservation areas (Figure 1). We found, based on the pre- felling data, that the forest stands of ACOSA are some of the most biomass rich areas of Costa Rica while, those of ACAHN are some of the most biomass poor. Supporting the findings of Stegen et al. [46], a comparison of conservation area level wood density and EAGB trends suggests that wood density alone cannot explain regional EAGB variability. For example, despite having one of the highest mean wood density values, ACAHN has one of the lowest mean EAGB values. The variation of EAGB between NFMPs was very high, as indicated by the large standard deviation of EAGB among conservation areas (Table 2). Our findings highlight the need for a better understanding of both the environmental and human variables influencing the distribution of EAGB across spatial scales. For example, studies have found that forest fragmentation has a strong negative impact on AGB and AGC (aboveground carbon) due to a significant increase in the mortality of large trees near forest edges [47],[48]. A greater comprehension of the factors controlling EAGB distribution will allow for the production of more reliable EAGB maps at local, regional, and national scales. Comparing the AGB estimates of our study to published estimates reveals that, in the regions of Costa Rica where EAGB has been previously sampled, our NFMP based approach produced a similar range of values. A study conducted at the La Gamba biological station in ACOSA reported the EAGB of trees with a DBH ≥ 30 cm was 218.46 ± 29.01 Mg ha^-1^[49]. After considering one standard deviation from the mean, the EAGB determined from NFMPs (173.47 ± 60.23 Mg ha^-1^) overlaps with the published estimates of Hofhansl et al. [49]. At ACCVC's La Selva biological station, Clark and Clark [17] found the density of large trees ranged from 4.7 to 10.1 stems ha^-1^ and the EAGB of large trees (DBH ≥ 70 cm) ranged from 22.6 to 55.4 Mg ha^-1^. The analysis of ACCVC NFMPs found values comparable to those reported by Clark and Clark [17], with a large tree density of 5.27 ± 3.39 tree ha^-1^ (census) and 6.71 ± 4.02 tree ha^-1^ (inventory) and a large tree (DBH ≥ 70 cm) EAGB of 27.53 ± 18.06 Mg ha^-1^ (census) and 34.84 ± 22.43 Mg ha^-1^ (inventory). This indicates a positive aspect in using NFMP data for assessing biomass and carbon [18].

Over half of the variation in EAGB across the five conservation areas sampled in this study was explained by the density of large trees. Although the strength of this predictive variable was approximately 20% less than the value reported by Slik et al. [32], our study supports the conclusion that large tree density accounts for a significant portion of EAGB variability across tropical regions. Additionally, we found that the patterns of EAGB and large tree density matched among conservation areas, demonstrating the importance of large trees as drivers of regional EAGB differences across Costa Rica.

Despite such a great amount of EAGB variability across NFMPs, a weak relationship was found between the EAGB of trees with a DBH < 60 cm but ≥30 cm and the EAGB of trees with a DBH ≥ 60 cm. If these results are consistent throughout other components of forest biomass, they indicate that models developed to estimate unmeasured portions of forest biomass based solely on the EAGB of measured forest components (e.g. [18]) may lead to an underestimation of the variability of forest biomass across the tropics. Future studies aiming to identify key variables that best explain how EAGB is distributed throughout different DBH classes and other forest stand components (e.g. lianas, coarse woody debris) could greatly improve the accuracy of AGB estimates (particularly in smaller trees) and in turn, the effort required to conduct large scale studies.

Our results show that the plot based sampling methodology of NFMPs (i.e. the inventory) tends to overestimate EAGB when compared to EAGB values calculated from the census of an entire forest stand. Houghton et al. [50] reported a similar result, finding a weak negative correlation between area sampled and EAGB. Both our results and those of Houghton et al. [50] indicate that the total area sampled may have important negative impacts on tropical AGB estimates. An additional source of the differences found may be explained by the location and distribution of inventory plots within forest stands. Although NFMP protocols specify that plots be randomly placed, we found a significant bias towards higher EAGB regions of the forest stand. As we do not have data to fully resolve the reason behind the bias, we hypothesize that it may be explained by a desire to achieve a higher economic outcome from the NFMP (i.e. to log a greater number of species and trees) and/or to reduce sampling effort (i.e. the placement of plots in more convenient areas of the forest stand). No matter the reason behind the bias, this finding brings to light the need to evaluate the sustainability of forest management practices in Costa Rica. If inventories are not only overestimating the number and EAGB of trees with a DBH ≥ 60 cm, but the number and EAGB of trees within the 30 cm to 60 cm DBH range, they may also be overestimating the capacity of forests to recover after a selective logging event [51].

### The uncertainty of EAGB

Our uncertainty analysis explored how incorporating wood density values at different scales in allometric models will introduce different amounts of uncertainty into a tree's estimated AGB. We found that more general stand level and regional wood densities can lead to uncertainties in the EAGB of a single tree between 27% and 28%. Further, we investigated how much the uncertainty of a tree's EAGB will increase when using a genus versus a species level wood density average. Although the 4% increase in uncertainty reflects the taxonomic composition of the forest stands sampled in this study, we believe that future work should consider this source of uncertainty when reporting EAGB estimates. Particularly, studies should pay greater attention to species and genera that exhibit high levels of wood density variability in the tropics (refer to Chave et al. [52] for a list of genera). The impact of wood density variability on EAGB uncertainty will be the greatest when (1) the species and/or genera sampled are highly variable and (2) the highly variable species and/or genera compose a notable portion of a forest stems and the wood density values incorporated into allometric models. When moving from the tree level to the plot level, the uncertainties introduced by measurement errors (wood density or DBH) decrease as the number of trees sampled increases. Errors introduced by the allometric model, on the other hand, can be either an issue of accuracy or precision [18],[33]. If the allometric error is consistent, regardless of sample size, an accuracy error is present (i.e. the allometric model does not apply to or represent the given area). If the allometric error differs between trees and decreases with an increase in sample size, it is a precision error. As this study does not include direct tree biomass measurements collected via destructive sampling, the accuracy of the allometric model could not be addressed [10]. Therefore, in our uncertainty analysis we simulated the impact of random allometric model error on EAGB to evaluate the precision of the allometric models, finding that allometric model errors introduced 37% uncertainty to tree level EAGB values. It is important to note that the development of Chave et al.'s [25] wet forest model included samples collected in Costa Rica (La Selva) while the moist forest equation did not. Future studies should evaluate the accuracy and applicability of these models in Costa Rican forests, particularly those that did not incorporate any Costa Rican data during their development.

Allometric model selection is an important source of error that was not directly addressed in this study. Several studies comparing the results of allometric models have shown they produce vastly differing results (e.g. [33],[53]-[55]). Further, Pelletier et al. [55] demonstrated that two different allometric models can result in estimated annual emissions from deforestation that differ by up to 48%. Given the potential impact of model selection on forest biomass estimates and in turn, the success of international mechanisms such as REDD+, future studies will need to consider this source of uncertainty. In this study we consciously selected the Chave et al. [25] models as they fulfill several key criteria (the inclusion of wood density, representation of a large DBH range, and development from a large sample size).

Feldpausch et al. [56],[57] found that an additional source of error in AGB estimates results from the exclusion of height as a predictor variable in allometric models. Regardless of this finding, Feldpausch et al. [57] also reported that the decrease in AGB estimation occurred only in smaller DBH classes (≥40 cm) and not larger ones. As our study only includes trees with a DBH ≥ 30 cm, we believe that the exclusion of height in the models applied to estimate AGB will not greatly increase the error of the reported values.

## Conclusions

Our study presents the most spatially rich analysis of ground level EAGB data in Costa Rica to date. Using data from forest management inventories, we found that the EAGB within and among five Costa Rican conservation areas is highly variable. Further, we detected bias in the NFMPs towards biomass rich areas of the forest stand, demonstrating the need to assess the sustainability of Costa Rican forest management practices. Expanding upon this finding, if ecological plots are also being preferentially located in more easily accessible areas of forest stands, studies may not be accurately capturing the EAGB of tropical forests [10].

Despite the potential taxonomic issues and missing DBH classes within NFMPs, our EAGB values were comparable to those reported in the scientific literature, supporting their inclusion in future EAGB assessments. Currently, the ground level data used to produce large-scale AGB and aboveground carbon maps is predominantly collected from ecological studies. Although this data is detailed and systematic in nature, ecological plots tend to sample either protected regions of the landscape or areas subject to a lesser amount of human impact. Forest management data, on the other hand, covers a considerable portion of the tropics and represents forests that are being greatly impacted by anthropogenic activities. In fact, Asner et al. [9] found that human activity was the greatest driver of AGB and aboveground carbon in the forests of Panama, highlighting the need measure the impact of humans on the variability of AGB and AGC across the tropics. Combining commercial logging inventories with ecological plots will provide a more representative ground level dataset for the calibration of the models and remotely sensed data used to estimate AGB and aboveground carbon at regional and national scales. Additionally, it is the non-protected areas of the tropics that offer the greatest opportunity to reduce rates of deforestation and forest degradation. Therefore, by improving our knowledge on the variability of aboveground carbon and AGB through forest management data, studies can better support the REDD + mechanism and the sustainable management of the rich natural resources of the tropics.

## Authors’ original submitted files for images

Below are the links to the authors’ original submitted files for images. Authors’ original file for figure 1 Authors’ original file for figure 2 Authors’ original file for figure 3 Authors’ original file for figure 4 Authors’ original file for figure 5 Authors’ original file for figure 6 Authors’ original file for figure 7
