# Supplementary figures and images for: A wood density and aboveground biomass variability assessment using pre-felling inventory data in Costa Rica
Source: Carbon Balance Manag. 2014 Sep 17;9:9. doi: 10.1186/s13021-014-0009-y (PMC4165877; doi:10.1186/s13021-014-0009-y)

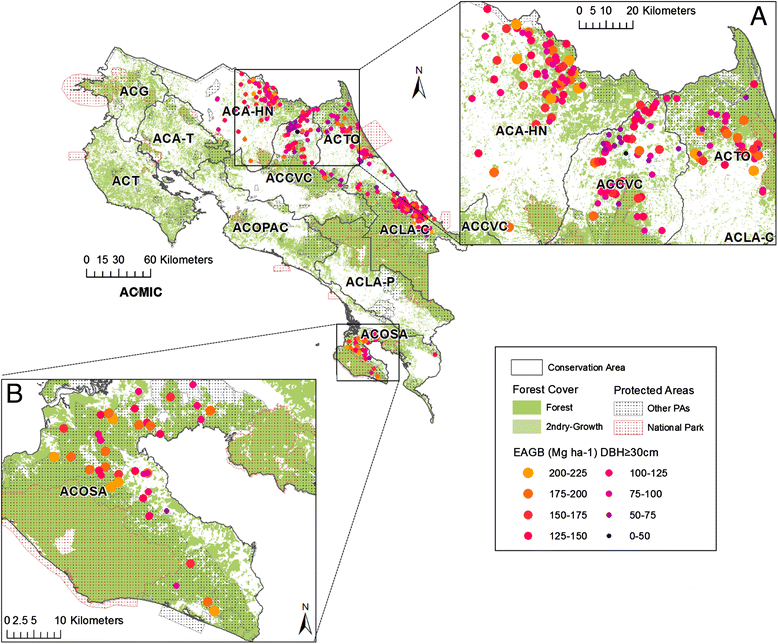

Supplement: Supplementary file 1 — Authors’ original file for figure 1 [file 13021_2014_9_MOESM1_ESM.gif]

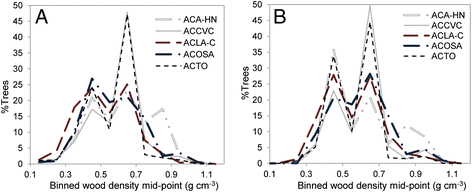

Supplement: Supplementary file 2 — Authors’ original file for figure 2 [file 13021_2014_9_MOESM2_ESM.gif]

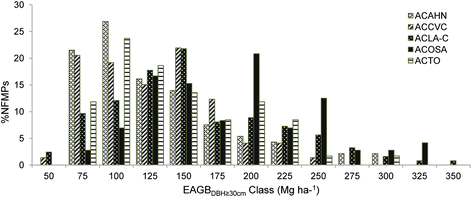

Supplement: Supplementary file 3 — Authors’ original file for figure 3 [file 13021_2014_9_MOESM3_ESM.gif]

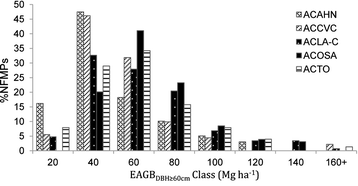

Supplement: Supplementary file 4 — Authors’ original file for figure 4 [file 13021_2014_9_MOESM4_ESM.gif]

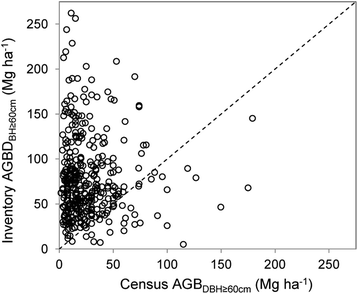

Supplement: Supplementary file 5 — Authors’ original file for figure 5 [file 13021_2014_9_MOESM5_ESM.gif]

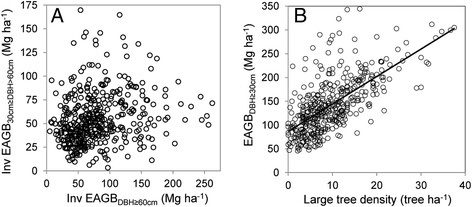

Supplement: Supplementary file 6 — Authors’ original file for figure 6 [file 13021_2014_9_MOESM6_ESM.gif]

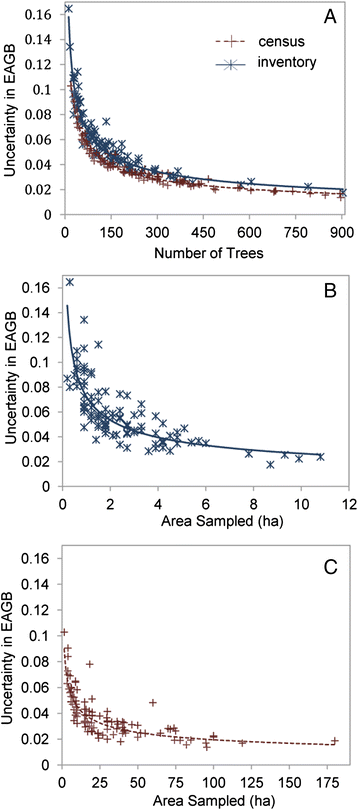

Supplement: Supplementary file 7 — Authors’ original file for figure 7 [file 13021_2014_9_MOESM7_ESM.gif]
